# Prioritisation of Provinces for African Swine Fever Intervention in South Africa through Decision Matrix Analysis

**DOI:** 10.3390/pathogens11020135

**Published:** 2022-01-22

**Authors:** Leana Janse van Rensburg, Mary-Louise Penrith, Eric M. C. Etter

**Affiliations:** 1 Department of Production Animal Studies, Faculty of Veterinary Sciences, University of Pretoria, Onderstepoort 0110, South Africa; eric.etter@cirad.fr; 2 Directorate Animal Health, Department of Agriculture, Land Reform & Rural Development of South Africa, Pretoria 0001, South Africa; 3 Department of Veterinary Tropical Diseases, Faculty of Veterinary Sciences, University of Pretoria, Onderstepoort 0110, South Africa; marylouise@vodamail.co.za; 4 CIRAD, UMR AnimalS Territories Risks Ecosystems (ASTRE), 97170 Petit Bourg, France; 5 ASTRE, University Montpellier, CIRAD, INRAE, 34070 Montpellier, France

**Keywords:** African swine fever, analytic hierarchy process, multi-criteria decision analysis, pigs

## Abstract

South Africa has experienced an increase in the number of African swine fever (ASF) outbreaks in domestic pigs in the last ten years. Intervention will be needed in the form of control and prevention strategies to minimise the impact of this disease in the country. The aim of this study is to prioritise which provinces resources should be allocated to for ASF intervention strategies, based on the risk factors identified as pertinent in South Africa. A multi-criteria decision analysis approach was followed using an analytic hierarchy process (AHP) method to determine the perceived risk of ASF outbreaks in domestic pigs per province. Nine risk factors applicable to the South African context were identified from literature. Data on the presence of these risk factors per province were collected from records and by means of a questionnaire. The risk factors were weighted by means of an AHP. The decision matrix determined that ASF intervention and prevention resources should be focused on Mpumalanga, Free State and Gauteng provinces in South Africa. Specific intervention strategies should be focused on the confinement of pigs, swill-feeding of pigs and buying/selling of pigs at auctions through a participatory approach with stakeholders.

## 1. Introduction

African swine fever (ASF) remains one of the chief limitations for pig production in Africa, causing high mortality with no available vaccine or means of treatment [1,2,3]. The ASF virus is the sole virus in the *Asfarviridae* family and infects wild suids and domestic pigs [4]. Ticks of the *Ornithodoros moubata* complex are the natural biological vector of the ASF virus, and all investigated species of *Ornithodoros* can also act as biological vectors that can maintain and transmit the virus for several years [5,6,7]. Primarily labelled as an African disease following its first description in 1921, it has since become one of the main transboundary diseases of concern in pigs, most recently affecting Europe [8], Asia [9] and more recently, Dominican Republic and Haiti in the Caribbean region [10].

South Africa has an ASF sylvatic cycle (between soft ticks and warthogs) present in a defined area in the north of the country, with only occasional spill-over to domestic pigs, usually due to biosecurity breaks [11,12,13]. South Africa has unfortunately experienced an increase in the number of ASF outbreaks in domestic pigs in the last ten years [10], with epidemics in 2012, 2016/17 and 2019/20 by means of domestic pig cycle transmission (domestic pig-to-pig transmission) [10,14]. This development of the domestic pig cycle of ASF in South Africa indicates that the country risk profile has changed [15]. As the risk of disease is not evenly distributed throughout a country, it makes sense to focus resources in areas where they would be most effective. An example is risk-based surveillance, where fewer animals need to be tested to confirm presence of the disease [16]. 

South Africa is divided into nine provinces. In South Africa, even though animal disease control is listed as a concurrent national and provincial function in Schedule 4 of the Constitution of the Republic of South Africa, 1996, veterinary services are listed as an exclusive provincial function in Schedule 5 of the Constitution. This implies that each provincial veterinary service would need to identify which animal diseases should be prioritised in their province for prevention and management strategies. The national veterinary authority of South Africa and industry stakeholders would benefit from the knowledge of which provinces should be prioritised for engagement and assisted in ASF disease management strategies.

In a situation where multiple factors must be considered to achieve objectives, a multi-criteria decision analysis (MCDA) can be used to come to a decision. All factors do not contribute equally to the ultimate decision, and thus the weighting of these criteria can be performed by using an analytic hierarchy process (AHP). In an AHP model, both physical and social criteria can be taken into account in the weighting process by measuring the criteria relatively in a hierarchical structure [17]. MCDA has been used in Southern Africa in many different spheres, such as disease risk analysis, health care and mining sectors [18,19,20,21,22].

The aim of this study is to identify which risk factors for ASF are of primary importance in South Africa and should be addressed by intervention strategies, as well as which provinces need to be prioritised to implement these strategies in order to manage and prevent further outbreaks of ASF in domestic pigs in the country.

## 2. Results

The risk factors that were identified for this study and the motivation for inclusion are shown in Table 1.

### 2.1. Data Collected

Data were collected for the presence of each of the risk factors per province (Table 2). 

**Figure 1:**
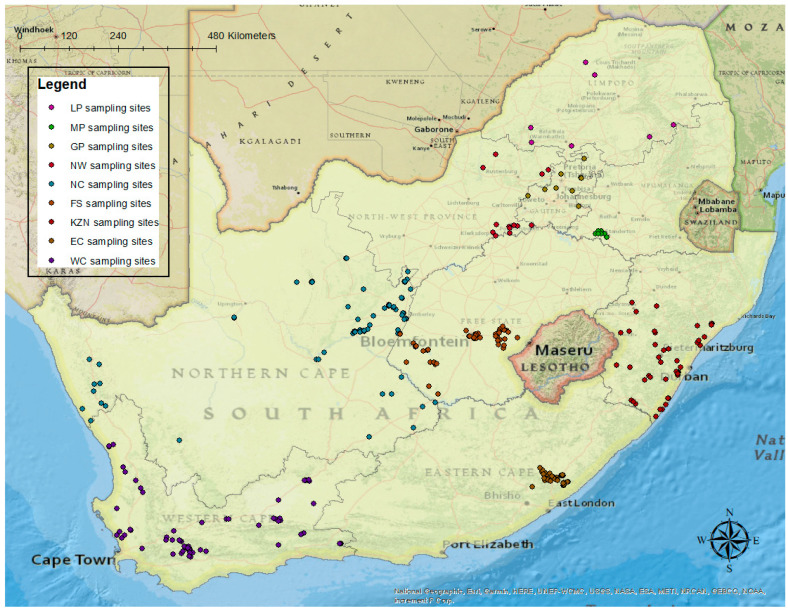
Location of questionnaire responses received.

There were 466 questionnaire responses received in total (Figure 1), which exceeds the determined minimum sample size, resulting in some responsiveness percentages exceeding 100%. Some of the questionnaires were not fully completed, resulting in fewer responses for some of the risk factors than the total number of questionnaires received. 

In the case of the Eastern Cape province, the questionnaire had been adapted for their awareness activities, which resulted in three of the risk factors being omitted from the responses (marketing, home slaughter and knowledge). For calculation purposes, a value of 0 was utilised for these three risk factors in the Eastern Cape.

Of the 466 pig herds represented, the median herd size of the respondents was ten, with a minimum herd size of 1 pig and the largest herd representing over 4000 pigs.

### 2.2. Analytic Hierarchy Process

Of the thirty-two experts approached to complete the pairwise comparison form, ten returned responses. Of these, seven were found to be consistent (CR < 0.2). The criteria weights of the consistent experts were then averaged to determine the final weighting of each risk factor (Table 3). 

### 2.3. Final Calculation of Priority

**Figure 2:**
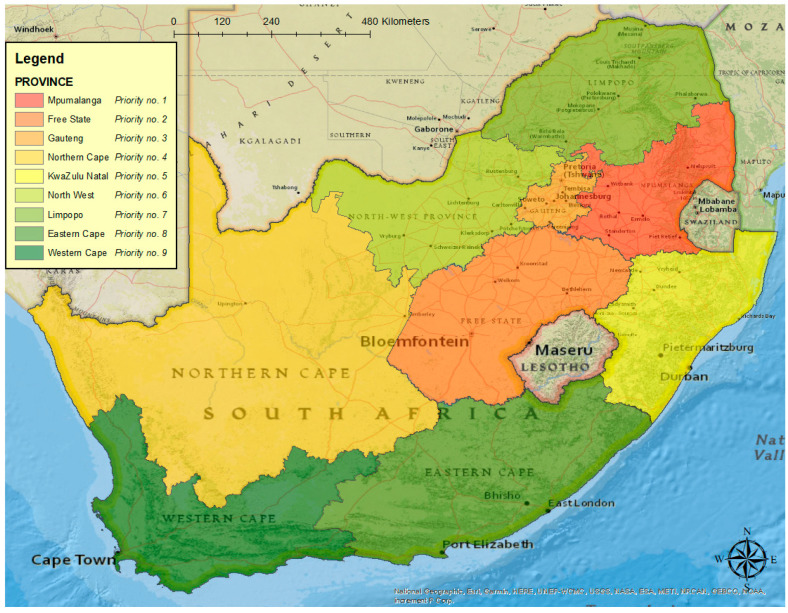
Map indicating which provinces in South Africa should be prioritised for ASF interventions.

Following the standardisation of the risk factors, a decision matrix was compiled with the standardised values, and the criteria weights and priority scores were calculated (Table 3). The province with the highest score was found to be Mpumalanga, followed by Free State and Gauteng (Figure 2). The province with the lowest score was found to be the Western Cape.

## 3. Discussion

As veterinary services in South Africa are divided into provincial units, it is sensible in this context to determine in which provinces ASF should be prioritised. Deciding which province should be prioritised for ASF intervention is not straightforward, as there are many factors which need to be considered, and not all of these factors are equally important. A MCDA using an AHP method is one way to analyse the collected data and be able to come to an overall decision, even when the data are complex and include ordinal data. [17,20,22]. However, it can be modelled in different ways and this study represents only one method, with the specific aim to assist the veterinary services and industry stakeholders of South Africa to prioritise the distribution of ASF intervention and prevention resources.

Based on the data collected on risk factor occurrence in the respective provinces, and using the weight per risk factor, it was determined that the province that needs to be prioritised the most for ASF intervention and prevention is Mpumalanga, with Free State and Gauteng following. Prior to ASF domestic pig cycles occurring in South Africa (2012), awareness of the disease was only focused in the legislated ASF controlled areas based on the endemic sylvatic cycle. This may have led to pig owners outside of the controlled areas not being aware of the risk of ASF, only viewing warthogs as potential sources of infection or thinking they were safe due to their location. Awareness outside of the controlled areas is essential. Not only has the epidemiology in South Africa changed, but the previous zoning is no longer as relevant in predicting the risk of exposure to the virus. It is therefore recommended to start with intensive awareness and ASF prevention activities, as well as increased surveillance in these three provinces.

Due to the absence of responses on the marketing, home slaughter and knowledge risk factors for the Eastern Cape, it can be assumed that there is an underestimation of this province’s risk priority. This can be demonstrated by the fact that if only the six factors for which data are available are used, the end score is much higher (0.45). The completed questionnaires from Mpumalanga and the Eastern Cape also appear to have originated from clustered locations within the provinces. This may be because most of the pigs are located in these areas, or that provincial officials have deemed those areas to be most in need of ASF awareness, but this clustering can potentially lead to a shortcoming in the representativeness of the responses for the whole province. Other publications on pigs in the Eastern Cape rather suggest a widespread distribution of pigs in the province [45,46].

Some of the risk factors selected are crude measures, and the specificity could be greatly improved if more data were available. Instead of using pig numbers per province, it would have been even more significant if one had more information on the number of pigs kept per level of biosecurity. Similarly, instead of only the warthog presence, more specific data on the distribution of soft tick vectors (especially those harbouring the virus), would have increased the significance. Further research in this regard is recommended.

For the standardisation of certain risk factors, i.e., number of outbreaks, pig population and province responsiveness, there could be many potential ways to approach the matter, but the aim for these risk factors was to try to divide the provinces into three roughly equal categories of low, medium and high risk. Warthog presence or absence was noted for this particular risk factor. This was found to be a practical approach, and can be refined in future studies.

For questionnaire administration, since there was no exhaustive list of pig owners, the owners could not be randomly selected, but provincial officials selected owners they were aware of or came into contact with during the awareness campaign, which focused more on non-commercial pig farmers. The sensitivity of the data on the presence of the risk factors measured by means of the questionnaire would be considerably increased if a list of pig owners was available, and sampling could also be better stratified within the provinces. It is recommended that a census of pig owners be carried out, not only to ensure better coverage of ASF awareness activities, but also to improve the planning of surveillance activities, as well as forming the first step in developing a traceability system. 

For the AHP conducted in this study, experts with a CR < 0.2 were included in the calculations. Saaty [47] suggested using a cut-off of 0.1; however, with the inclusion of nine risk factors to be compared pairwise (resulting in 36 comparisons), more inconsistency was to be expected. Only three experts had a CR < 0.1; if only these had been used, it would have limited representativeness, but using only the data from these three experts resulted in the same three risk factors with the highest weighting, as well as the same first-priority province as that which was found using the data from the seven experts (data not shown). A second round of expert elicitation could have been organised to reduce the irrelevance of the pairwise comparison for experts having a CR > 0.1, but this was not possible due to time constraints.

Using the AHP, the experts determined that in the South African context the most important risk factors were pigs not being kept confined, pigs being fed untreated swill potentially containing meat and the buying and/or selling of pigs at auctions (Table 3). By conducting a ranking of the risk factors by means of a pairwise comparison, it is hoped that they will each be ranked on their own merit and not be influenced by the frequency of risk factor occurrence in the local context (with more frequent risk factors being seen as more important). For future studies, it may be advisable to include experts not familiar with the local context for the weighting of the risk factors, to prevent any such possible bias.

Specific attention and intervention strategies should be focused on the confinement of pigs, swill-feeding of pigs and use of livestock auctions through a participatory approach with stakeholders. These three main risk factors are governed by human behaviour and are thus preventable by good biosecurity practices. Good biosecurity practices should be the main focus of the ASF prevention strategies that require implementation. This is similar to what has been proposed for the control of ASF in Europe [8]. Confining pigs to a property where the owner is in control of what the pigs are exposed to would be ideal, and is essential for semi-commercial-to-commercial pig farmers. This has been found effective in the recent outbreaks in Timor-Leste [48], where fences were made from corrugated metal roof sheeting. However, especially in resource-limited communities, it would not be socially acceptable to prohibit free-roaming pig systems [29]. In many instances, pigs are kept by the poor because they do not necessarily have to be fed, but can scavenge for themselves and/or convert kitchen waste into edible protein [15]. For these subsistence farming systems, it would be paramount that these communities are targeted for engagement on awareness of potential disease transmission routes and promotion of the buy-in of all pig owners in an area to prevent the spread of ASF [49]. Identification of potential ASF sources in these areas needs to be highlighted and appropriate measures on how to approach these risks should be discussed with the community using participative approaches [50]. These prevention and control measures should be perceived as positive by pig owners, and should be innovative and increase the role of the community in ASF control [29,37]. In South Africa, livestock auctions are live-animal markets where pigs are sold in lots to the highest bidder via an auctioneer. The participation in auctions as a marketing strategy is often used by small-scale pig farmers, as it is an easy way to market a small—and perhaps inconsistent—number of animals. Intervention strategies for this risk factor could look at assisting small-scale pig farmers with marketing options, such as forming cooperatives [42]. Biosecurity at auction premises, with full traceability of animals, health checks before off-loading and health declarations from the herds of origin are also recommended as intervention strategies, with the engagement and cooperation of auctioneers being paramount. 

As was seen in previous domestic pig cycle ASF outbreaks in South Africa, due to these anthropogenic factors involving the movement of pigs and their products, outbreaks have not spread contiguously from one area to another but have rather been seen to “jump”. This has also been seen in Russia and China [35,51,52] and emphasises that pig owners should not become complacent regarding the implementation of biosecurity measures if there have been no reports of outbreaks in their immediate vicinity. This was demonstrated very recently in South Africa, where outbreaks occurred in the Western Cape province [10], which were suspected to have spread due to anthropogenic activity from the Eastern Cape province.

## 4. Materials and Methods

An MCDA approach was followed to determine the perceived risk of ASF outbreaks in domestic pigs per province. This entailed searching literature for risk factors associated with ASF outbreaks in domestic pigs. The risk factors were then weighted according to their relative importance by experts by means of an AHP, in order to determine which risk factors are seen as most relevant to the South African situation. Data on the presence of the selected risk factors per province were then collected, both from available records [13,23,25,27] and by means of a questionnaire. Finally, a decision matrix was compiled using the collected data and the weighting of the risk factors, in order to determine which provinces should be prioritised for ASF intervention due to the risk of ASF outbreaks in domestic pigs.

### 4.1. Risk Factor Identification

Risk factors that lead to the introduction and spread of ASFV in domestic pig populations were identified from available literature and consulted via the University of Pretoria library and their internal research engines. The risk factors were discussed with the South African veterinary services and representatives of the pig industry of South Africa to confirm applicability to the local context. 

### 4.2. Provincial Data Collection

Data on the provincial presence of these factors were collected by means of available records and a questionnaire administered to pig keepers throughout South Africa as part of an ASF awareness drive by the veterinary services of South Africa.

The number of previous outbreaks (2000–2020) per province was obtained from the South African Department of Agriculture, Land Reform & Rural Development (DALRRD) disease database [23] and OIE WAHIS [10]. The number of pigs present per province was obtained according to the report on the 2016 Agricultural Household of the Community Survey [25]. The presence of warthogs in the province was obtained according to the red list of mammals of South Africa, Swaziland and Lesotho [27]. The data on the other risk factors were collected by means of the questionnaire administered. Provincial veterinary officials, as well as business development managers employed by the South African Pork Producers’ Organisation, administered the questionnaires to pig keepers encountered during an ASF awareness drive.

To determine the required sample size for the questionnaires in a large population (the sampling frame being primarily the smaller-scale pig keepers of South Africa), the expected frequency of the presence of risk factors was taken at 50% to maximise the sample size. A 95% confidence interval with a desired absolute precision of 5% was utilised.

The formula used was adapted from Thrusfield [53]:

(1) $$n=\frac{1.96^{2}\;P_{exp}\left(1-P_{\exp}\right)}{d^{2}}$$

where n = required sample size, $P_{exp}$ = expected frequency of the presence of risk factors and d = desired absolute precision.

This determined that a minimum of 384 questionnaire responses were required in total. This number was then stratified per province, according to the percentage of the total number of pigs in South Africa present in that province [25]. The provincial veterinary services were then requested to perform a minimum number of questionnaires with pig owners during their ASF awareness drive. Maps for this study were created using ArcGIS^®^ software by Esri. (ArcGIS® and ArcMap™ are the intellectual property of Esri and are used herein under license. Copyright © Esri. All rights reserved. www.esri.com).

The questionnaire included questions on whether pigs were confined at all times; what the pigs were fed; where pigs were obtained and marketed; and whether any pigs were slaughtered at home. The following guidelines were provided to the administrators of the questionnaires to determine the pig owner’s level of knowledge of ASF:

None: Does not know about ASF;

Poor: Has heard of ASF but does not know the clinical signs or how it is spread;

Reasonable: Knows about ASF and that it causes haemorrhaging and mortality;

Good: Knows about ASF and the clinical signs and that it is caused by a virus and how the disease is prevented.

### 4.3. Standardisation of Risk Factors

For each of the nine risk factors selected, rules for standardisation were determined and assigned a risk value between 0 and 1. For the data obtained from records, this was achieved by determining two or three categories which could be equated to a lower/higher risk of ASF outbreaks, thus dividing the values into roughly equal categories. The number of completed questionnaires received per province as a percentage of the minimum number requested was used for the “responsiveness” risk factor. For the data obtained from the questionnaires, the percentage of responses confirming the presence of the risk factors was utilised. 

The risk factors were standardised to assign a risk value between 0 and 1, as per the rules in Table 4.

### 4.4. Analytic Hierarchy Process

**Figure 3:**
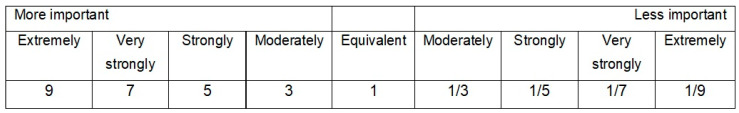
Simplified Saaty scale used for comparing risk factors with the AHP method.

For the AHP, risk factors were compared in a pairwise manner by experts, to determine a perceived hierarchy of importance. The form for pairwise comparison of the risk factors was compiled on Google Forms, and the link sent via email to experts, in order to determine the relative importance of the risk factors in the South African context. Experts were selected based on relevant experience and knowledge on ASF and the local situation in South Africa. The experts selected were all members of the South African ASF working group (33) and included officials from DALRRD, provincial veterinary officials, researchers and academics. The method utilised was adapted from the AHP [47]. The experts were sent a questionnaire to complete on Google Forms, which compared two risk factors at a time on the Saaty scale (Figure 3). Nine is the maximum number of factors recommended to be used in an AHP, as an increase in number of factors compared leads to decreased consistency in the comparisons.

The criteria weight of each risk factor per expert was calculated by converting the different comparisons two at a time into a pairwise comparison matrix, then normalising the values in this matrix and finally taking the mean of the normalised values.

Each expert’s matrix was then evaluated for consistency by calculating the consistency ratio (CR):

(2) $$CR=\frac{CI}{RI}$$

where the Consistency Index (CI)

(3) $$CI=\frac{\left(\lambda \max-n\right)}{\left(n-1\right)}$$

with n the number of risk factors in the comparison and

(4) $$\lambda \max=\underset{i,j}{\sum }\left(sum_{i}*weight_{j}\right)$$

where *sum* is the sum for each column *i* of the numerical matrix and *weight* is the sum for each row *j* of the normalised matrix.

The Random Index (RI) is dependent on the number of risk factors and is 1.45 for nine factors [47].

For this study, if the CR was more than 0.20, there was an indication that the prioritisation was not consistent enough to be of value, and the matrixes from these experts were excluded due to the inconsistency. 

The criteria weights of the consistent experts were then averaged to determine the final criteria weight for each risk factor.

### 4.5. Final Calculation of Priority

A decision matrix was compiled with the standardised values per risk factors per province. These values were then multiplied with the criteria weight for that risk factor as determined through the AHP. The sum of the weighted values for all nine risk factors per province determined the final risk value for that province.

## 5. Conclusions

With the increase in ASF outbreaks in domestic pigs in South Africa, in a historically “ASF-free” area of the country, intervention will be needed in the form of control and prevention strategies in order to minimise the impact of this disease in the country. However, as with most animal disease control authorities worldwide, resources are limited. It is therefore sensible to establish where resources should be dedicated in order to address the highest risk areas. By means of an MCDA using the AHP method, it was found that ASF intervention and prevention resources should be focused on the Mpumalanga, Free State and Gauteng Provinces in South Africa, and more investigation is warranted for the Eastern Cape Province. Specific attention and intervention strategies should be focused on the confinement of pigs, swill-feeding of pigs and buying/selling of pigs at auctions through a participatory approach with stakeholders.

## Figures and Tables

**Table 1:** Identified risk factors and motivation for inclusion.

Risk Factor	Reason for Inclusion	References
Number of ASF outbreaks reported in that province from Jan 2000 to Oct 2020	Previous outbreaks in the area may indicate that a source of ASFV could still be present in the area.	[10,11,14,23]
Number of pigs in the province	In order for ASF outbreaks to occur, it stands to reason that domestic pigs should be present, and that the more pigs, the higher the risk of an outbreak there is.	[19,24,25]
Whether warthogs are present in the province	Although very simplified, if wildlife reservoirs of ASF are present in the province, they could serve as a potential source/maintainer of virus.	[12,24,26,27]
Responsiveness to ASF questionnaire request per province	This can give a crude indication of the current priority of ASF prevention (as this was combined with an ASF awareness campaign) in the particular province.	[1,28]
Pigs not kept confined	This was found to have played a significant role in previous ASF outbreaks in both South Africa and other countries due to owners having no control over what the pigs come into contact with.	[19,29,30,31,32,33]
Feeding of uncooked swill potentially containing meat products	This was found to have played a role in previous ASF outbreaks in both South Africa and other countries due to the ASF virus being able to survive well in a proteinaceous environment.	[2,14,19,32,34,35,36,37,38,39,40,41]
Buying and/or selling at auctions	This was found to have played a role in previous ASF outbreaks in both South Africa and other countries due to the mixing of pigs of various origins, including some which may be at auction due to panic selling that occurs once pigs start dying.	[1,14,34,42]
Practising home slaughter	When pigs are informally slaughtered, there is no meat inspection performed to detect signs of ASF. Furthermore, households slaughtering pigs often provide meat to neighbours or sell the meat in the local community, which may contribute to the spread of disease. Disposal of the remains also presents problems, especially in areas with free-roaming pigs.	[1,24,33,36,37,40,42,43,44]
Poor knowledge of ASF	Where a pig keeper’s knowledge of ASF is poor to none, no measures are implemented to prevent the entry of ASF into the pig herd.	[34,44].

**Table 2:** Results from data collection on presence of ASF risk factors per province.

Province	Outbreaks (2000–2020)	Number of Pigs	Warthogs (Present /Absent)	Responsiveness (%)	Pigs Not Kept Confined (%)	Fed Uncooked Swill (%)	Use of Auctions (%)	Home Slaughter (%)	Poor ASF Knowledge (%)
Eastern Cape	7	536 366	Present	45.2 (57/126)	30.4 (17/56)	3.5 (2/57)	-	-	-
Free State	16	149 878	Present	228.6 (80/35)	24.1 (19/79)	48.1 (38/79)	27.8 (22/79)	53.8 (43/80)	78.9 (63/80)
Gauteng	17	141 145	Present	40.0 (14/35)	28.6 (4/14)	21.4 (3/14)	50.0 7/14)	21.4 (3/14)	21.4 (3/14)
KwaZulu-Natal	0	200 428	Present	88.0 (44/50)	9.1 (4/44)	38.6 (17/44)	13.6 (6/44)	50.0 (22/44)	60.0 (24/40)
Limpopo	25	135 112	Present	22.6 (7/31)	14.3 (1/7)	0 (0/7)	0 (0/7)	42.9 (3/7)	28.6 (2/7)
Mpumalanga	20	192 823	Present	64.4 (29/45)	20.7 (6/29)	34.5 (10/29)	93.1 (27/29)	0 (0/28)	79.3 (23/29)
Northern Cape	5	13 078	Present	3000.0 (120/4)	13.2 (29/119)	62.2 (74/119)	9.6 (11/115)	63.3 (74/115)	81.4 (96/118)
North West	7	127 702	Present	41.9 (13/31)	15.4 (2/13)	15.4 (2/13)	46.2 (6/13	23.1 (3/13)	7.7 (1/13)
Western Cape	0	105 417	Absent	377.8 (102/27)	18.6 (19/102)	15.7 (16/102)	3.0 (3/99)	77.5 (79/102)	83.3 (85/102)

**Table 3:** Decision matrix for prioritisation of provinces.

Risk Factors	Outbreaks (2000–2020)	Number of Pigs	Warthogs (Present/Absent)	Responsiveness (%)	Pigs Not Kept Confined (%)	Fed Uncooked Swill (%)	Use of Auctions (%)	Home Slaughter (%)	Poor ASF Knowledge (%)	
Average criteria weights	0.07585	0.05993	0.08451	0.05373	0.22103	0.18975	0.13964	0.05823	0.11735	
Province										**Score**
Eastern Cape	0.5	1	1	1	0.304	0.035	0	0	0	0.30992
Free State	1	0.5	1	0	0.241	0.481	0.278	0.538	0.788	0.49747
Gauteng	1	0.5	1	1	0.286	0.214	0.5	0.214	0.214	0.45536
KwaZulu-Natal	0	1	1	0.5	0.091	0.386	0.136	0.5	0.6	0.38317
Limpopo	1	0.25	1	1	0.143	0	0	0.429	0.286	0.31921
Mpumalanga	1	1	1	0.5	0.207	0.345	0.931	0	0.793	**0.58142**
Northern Cape	0.5	0.25	1	0	0.132	0.622	0.096	0.633	0.814	0.43039
North West	0.5	0.25	1	1	0.154	0.154	0.462	0.231	0.077	0.34139
Western Cape	0	0.25	0	0	0.186	0.157	0.03	0.775	0.833	0.23295

**Table 4:** Rules for risk factor standardistion.

Risk Factor	Number of Outbreaks	Number of Pigs	Warthog Presence	Responsiveness	Pigs Not Kept Confined	Fed Uncooked Swill	Use of Auctions	Home Slaughter	Poor ASF Knowledge
**Rule**	0 = 0 <10 = 0.5 >10 = 1	<140,000 = 0.25 140,000 to 190,000 = 0.5 >190,000 = 1	Present = 1 Absent = 0	>100% = 0 50–99% = 0.5 0–49% = 1	Percentage of responses received indicating the presence of this risk factor				

## Data Availability

The data presented in this study are available on request from the corresponding author.
